# Attack of the Clones: A Patient With Untreated Aplastic Anemia Presenting With Classical Paroxysmal Nocturnal Hemoglobinuria

**DOI:** 10.7759/cureus.34093

**Published:** 2023-01-23

**Authors:** Jose Rayas, Mariam Hassan, Rivers A Hock, Bryan Nguyen, Swathi Prakash, Adrian Rojas Murguia, Ilma Vahora, Javier Corral, Osvaldo Padilla, Fatma Dihowm

**Affiliations:** 1 Internal Medicine, Texas Tech University Health Sciences Center Paul L. Foster School of Medicine, El Paso, USA; 2 Hematology and Oncology, Texas Tech University Health Sciences Center Paul L. Foster School of Medicine, El Paso, USA; 3 Pathology, Texas Tech University Health Sciences Center Paul L. Foster School of Medicine, El Paso, USA

**Keywords:** immune hemolytic anemia, morbidity and mortality, bone marrow failure, acquired aplastic anemia, paroxysmal nocturnal hemoglobinuria (pnh)

## Abstract

Paroxysmal nocturnal hemoglobinuria (PNH) is an acquired X-linked, clonal hematopoietic stem cell disease. Patients with PNH may complain of vague symptomatology that contributes to the challenge of its diagnosis. This is especially true in the clinical context of a coinciding hematologic disorder. Aplastic anemia (AA) is an additional immune-mediated illness that results in the destruction of hematopoietic precursors and pancytopenia. The authors encourage screening for PNH clones in patients initially diagnosed with AA, treating underlying hematologic disease to prevent clonal expansion, and further research to investigate the effectiveness of eculizumab in an unusual “classical” PNH secondary to AA with hypercellular bone marrow.

## Introduction

Paroxysmal nocturnal hemoglobinuria (PNH) is rare, acquired X-linked disease with an estimated occurrence of one to five individuals per million worldwide [1-3]. A defect of the phosphatidylinositol glycan anchor (PIGA) gene leads to dysfunction of red cell membrane proteins, or glycosylphosphatidylinositol (GPI) anchor, that is responsible for protecting erythrocytes from complement-mediated destruction [2]. The GPI anchor proteins implicated here are CD55 (or decay-accelerating factor {DAF}) and CD59 (or membrane inhibitor of reactive lysis {MIRL}) [2,3]. The loss of their protective effect renders erythrocytes vulnerable to intravascular and extravascular hemolysis via the complement and reticuloendothelial systems, respectively [2,3].

PNH is characterized by a triad of hemolytic anemia, pancytopenia, and thrombosis [2,3]. Patients, however, may complain of vague symptomatology, such as fatigue, weakness, or chronic shortness of breath. This is diagnostically challenging, especially in the clinical context of a coinciding hematologic disorder. Aplastic anemia (AA) is an additional immune-mediated illness that results in the destruction of hematopoietic precursors and pancytopenia [4]. An idiopathic etiology accounts for approximately 65% of AA cases [5]. Fanconi anemia is the most common hereditary cause, and additional precipitators include viral hepatitis, medications, toxins, and ionizing radiation [5]. While antibody formation against red blood cells takes place, multipotent hematopoietic stem cells with PIGA mutation may escape these autoimmune attacks, encouraging clonal expansion in the bone marrow, and giving rise to PNH [4,6].

AA is a known risk factor of PNH which possesses significant morbidity and mortality. In patients with suspected PNH, rapid diagnosis and concomitant supportive care are critical. We herein report the case of a 29-year-old male with a history of untreated AA who was diagnosed with a “classical” PNH.

## Case presentation

A 29-year-old male with a past medical history of AA presented with generalized weakness and intermittent nausea for one week. His AA was diagnosed via a bone marrow biopsy performed in Afghanistan in 2018. Documentation of the same was provided. He denied having taken any medications, recreational drug use, or being diagnosed with a viral illness at the time. The patient denied a family history of blood or bone marrow-related disorders. The patient also reported having intermittent, nocturnal, gross hematuria for one year.

The physical examination was notable for pallor. There was no evidence of jaundice or splenomegaly. On admission, the patient had a low white blood count, red blood count, hemoglobin, platelets, and haptoglobin. He also had an elevated absolute reticulocyte count, serum creatinine without any history of chronic kidney disease, conjugated hyperbilirubinemia, creatine kinase, and lactate dehydrogenase. The initial laboratory values are depicted in Table 1 [7].

**Table 1:** Laboratory results from admission.

Tests	Results	Normal range
White blood count (WBC)	3.05×10^9^/UL	4.50-11.00×10^9^/UL
Red blood count (RBC)	2.50 × 10^6^/UL	3.50-5.50×10^6^/UL
Hemoglobin	7.4 g/dL	13.0-17.0 g/dL
Platelets	128×10^9^/UL	150-450×10^9^/UL
Absolute reticulocyte count	0.14×10^6^/UL	0.03-0.11×10^6^/UL
Haptoglobin	8 mg/dL	41-165 mg/dL
Serum creatinine	2.3 mg/dL	0.8-1.3 mg/dL
Total bilirubin	2.8 mg/dL	0.1-1.2 mg/dL
Direct bilirubin	1.7 mg/dL	<0.3 mg/dL
Creatine kinase (CK)	233 IU/L	25-200 IU/L
Lactate dehydrogenase (LDH)	4,187 IU/L	50-150 IU/L

**Figure 1:**
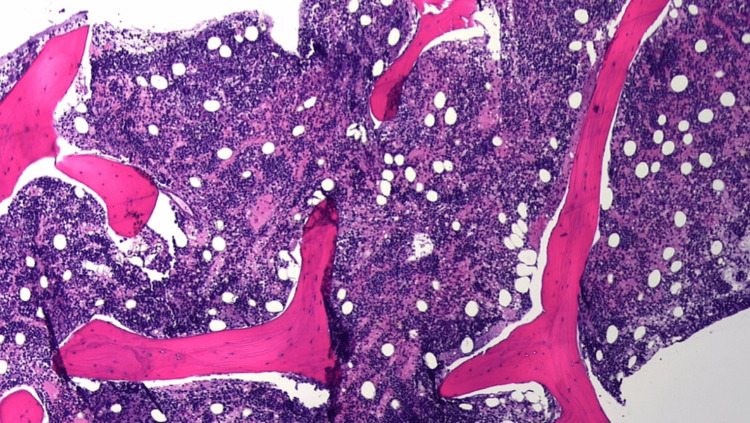
Bone marrow biopsy (4x magnification). The bone marrow biopsy at low magnification shows hypercellularity as noted by approximately 90% cellularity.

**Figure 2:**
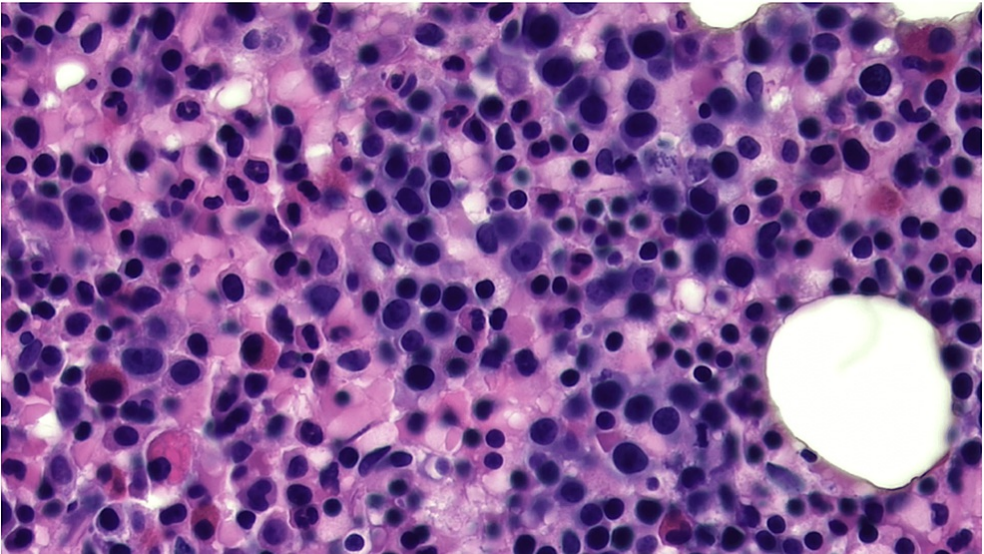
Bone marrow biopsy (20x magnification). This higher magnification shows numerous erythroid precursors as noted by variable-shaped, mononuclear cells with dense chromatin nuclear staining. These erythroid precursors show leftward-shifted maturation stages.

A peripheral blood smear showed 1+ schistocytes and 1+ spherocytes. A direct Coombs test and ADAMTS-13 were negative. An ultrasound of the abdomen showed normal spleen size and echotexture. A bone marrow biopsy and aspirate were performed to reveal a hypercellular bone marrow of approximately 90% cellularity (Figure 1). The hypercellularity is mostly due to increased erythroid precursors with leftward shifted maturational stage (Figure 2). An M:E ratio of approximately 1:10 is estimated, which is compatible with erythroid hyperplasia. Blasts were less than 5% and no dysplastic features were identified in either of the three lineages. Iron stores were low normal to mildly decreased with no ringed sideroblasts identified. Flow cytometry revealed CD59 deficiency in 9.85% of cells. No other phenotypic abnormalities were identified by flow cytometry.

The patient was initially started on cyclosporine, but no changes could be appreciated in his laboratory results or symptoms. He was subsequently started on eculizumab therapy with gradual improvement in weakness and nocturnal hematuria.

## Discussion

PNH can be diagnostically challenging due to the rarity of the illness, ambiguous symptoms, presence of comorbid conditions, and resource limitations in underfunded clinical settings. The authors speculate that, as a result of these factors, the incidence rate of PNH may be higher than what is commonly reported. Hence, more patients are diagnosed and treated later in the course of their disease [8].

A strong pathophysiological correlation exists between AA and PNH [4,9-13]. PNH, however, remained the top differential in this case for several reasons. As stated, our patient has a self-reported history of AA, one of the few known risk factors for PNH [9-13]. A thorough literature review highlights that 40% of patients with AA have a detectable clone of PNH cells during their disease course [14]. It is reported that 10-57% will have clonal expansion of PNH cells over time and later develop PNH [4,10-12].

The clinical spectrum of PNH is highly variable, with a hemolytic (classical PNH) variant on one end and an aplastic (AA/PNH syndrome) variant on the other [3]. A majority of classic PNH patients are expected to possess a normal or hypercellular marrow, with increased erythropoiesis, larger PNH clones, and less pronounced or absent peripheral cytopenias [15]. AA/PNH patients, on the other hand, have a hypocellular marrow [15]. Interestingly, the bone marrow biopsy of our patient revealed hypercellularity instead of an expected hypocellularity seen in patients with underlying bone marrow failure disease, thus suggesting active transformation of AA into a classical PNH picture [3,15].

Moreover, AA is typically treated with horse anti-thymocyte globulin (hATG) and cyclosporine [10]. Allogeneic bone marrow transplantation (BMT) is an alternative treatment for patients with severe AA [12]. BMT restores normal hematopoiesis and cures the disease in 60-80% cases [12]. Despite our patient having a history of AA, he denied having ever received immunosuppression that leads to clinical remission in a large proportion of patients [10]. Whether this was due to patient choice or resource limitations, the case exemplifies the health disparities present in the US-Mexico border region and accentuates the need to identify acute complications of chronic disease in this vulnerable patient population.

Eculizumab is offered as the standard of care for patients with classical PNH [16]. This terminal complement inhibitor is approved by the U.S. Food and Drug Administration (FDA) and functions by preventing the cleavage of complement protein C5, and thereby the formation of a terminal attack complex [16,17]. Eculizumab protects vulnerable red blood cells that lack CD55 and C59 from complement-mediated hemolysis [16,17]. It is a highly effective monoclonal antibody associated with a 70% reduction in the risk of thrombotic events and significant adverse vascular complications [18].

While classical PNH patients benefit from anti-complement therapy, there are limited publications to guide treatment in secondary PNH or AA/PNH patients. AA/PNH patients develop few, or if any, PNH-related symptoms and the development of clinical symptoms is related to clonal size [19]. Patients typically do not benefit from eculizumab as treatment is focused on the underlying bone marrow failure, thus our patient was initially started on cyclosporine [20,21]. A more recent UK-based case series unveiled patient outcomes among those with symptomatic PNH due to AA [21,22]. The case series proved that concurrent treatment with immunosuppressants and eculizumab offers no statistically significant improvement compared to immunosuppressive therapy alone [21]. Although exceptions occur, a small subset of patients with PNH and underlying BM disorder with relatively large clones or significant clinical hemolysis may benefit from complement inhibitor therapy, such as the eculizumab therapy that our patient was started on later [20]. Nevertheless, further investigation concerning the efficacy of eculizumab in secondary PNH is warranted [22].

## Conclusions

Ultimately, it is imperative that comorbid hematologic disease be screened for and diagnosed early on to prevent the acute complications of a chronic condition. Patients with AA are predisposed to expansion of PNH clones. PNH remains a debilitating, and even fatal, but treatable disease that demands timely care. Primary management in this patient population should be centered around treating the underlying bone marrow deficiency with immunosuppressive therapies including hATG and cyclosporine. Future studies should investigate the risks and benefits of adding anti-complement in AA patients who screen positive for PNH clones. An international blueprint may be devised to help expedite the diagnostic process and delineate treatment modalities of a PNH spectrum.
